# Endoglin in the Spotlight to Treat Cancer

**DOI:** 10.3390/ijms22063186

**Published:** 2021-03-20

**Authors:** Teresa González Muñoz, Ana Teresa Amaral, Pilar Puerto-Camacho, Héctor Peinado, Enrique de Álava

**Affiliations:** 1 Microenvironment and Metastasis Group, Department of Molecular Oncology, Spanish National Cancer Research Center (CNIO), 28029 Madrid, Spain; mtgonzalezm@cnio.es; 2 Molecular Pathology of Sarcomas, Institute of Biomedicine of Sevilla (IBiS), Virgen del Rocio University Hospital, CSIC, University of Sevilla, CIBERONC, 41013 Seville, Spain; mpilarpuertocamacho@gmail.com; 3 Department of Normal and Pathological Cytology and Histology, School of Medicine, University of Seville, 41009 Seville, Spain

**Keywords:** endoglin, tumor, microenvironment, targeted therapy, biomarker

## Abstract

A spotlight has been shone on endoglin in recent years due to that fact of its potential to serve as both a reliable disease biomarker and a therapeutic target. Indeed, endoglin has now been assigned many roles in both physiological and pathological processes. From a molecular point of view, endoglin mainly acts as a co-receptor in the canonical TGFβ pathway, but also it may be shed and released from the membrane, giving rise to the soluble form, which also plays important roles in cell signaling. In cancer, in particular, endoglin may contribute to either an oncogenic or a non-oncogenic phenotype depending on the cell context. The fact that endoglin is expressed by neoplastic and non-neoplastic cells within the tumor microenvironment suggests new possibilities for targeted therapies. Here, we aimed to review and discuss the many roles played by endoglin in different tumor types, as well as the strong evidence provided by pre-clinical and clinical studies that supports the therapeutic targeting of endoglin as a novel clinical strategy.

## 1. Introduction

**Figure 1:**
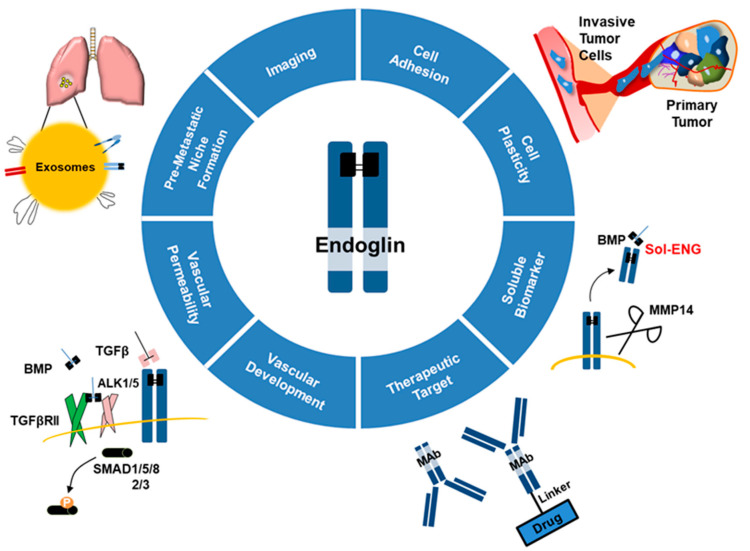
Endoglin in physiology and pathology. Scheme depicting how endoglin is associated with many physiological and malignant processes and might be a reliable biomarker and a therapeutic target depending on the cellular context. Transforming growth factor β (TGFβ), transforming growth factor β receptor II (TGFβRII), bone morphogenic protein (BMP), activin-like kinase (ALK), monoclonal antibody (MAb), matrix metalloproteinase 14 (MMP14), soluble endoglin (Sol-ENG).

Endoglin (ENG), also known as CD105, is a transmembrane receptor that was initially associated with neo-angiogenesis, yet it is currently known to be involved in several physiological and malignant processes (Figure 1). In this review, we describe how the molecular features of this protein make it an attractive therapeutic target, a reliable disease biomarker, and an important (yet somewhat controversial) player that modulates malignant phenotypes in cancer.

## 2. Endoglin: Molecular Features

The human gene encoding ENG is located on the long arm of chromosome 9 (9q34.11) [1]. The promoter of this gene lacks consensus TATA and CAAT boxes, yet it contains GC-rich regions and consensus motifs for specific modulators [2]. Indeed, the specificity protein-1 (Sp1) site at -37 seems to be necessary for the basal and transforming growth factor β (TGFβ)-induced transcription of ENG [3]. The gene product is a protein of 658 amino acids (aa), and it is arranged as a disulfide-linked homodimer [4,5] that establishes itself as an integral membrane glycoprotein [5]. As its glycosylation status may differ across tissues, the final molecular weight of ENG can vary between 90 and 95 kDa for each subunit [6]. The protein has three well-differentiated structures: (1) a 586 aa extracellular region (ectodomain), (2) a 25 aa hydrophobic transmembrane domain, (3) and a cytoplasmic tail of variable length [5,6].

The extracellular region contains an N-terminal signal peptide of 25 aa that is followed by a sequence of unknown function, an orphan domain (aa 26–330) that is enriched in N-glycosylation-prone residues, and a zona pellucida (ZP)-like domain (aa 349–576) [6,7,8]. The ZP domain is a ~260 aa cysteine (Cys)-rich sequence that is found in a family of proteins with similar structural features [7]. In particular, the Cys350 aa present in this part of the ectodomain is the Cys residue that participates in the disulfide-bond dimerization of the homomeric ENG [5,6,7,8,9]. Interestingly, the ZP domain of human ENG contains an arginine–glycine–aspartic acid (RGD) tripeptide that is typically found in extracellular matrix (ECM)-associated proteins, a sequence recognized by integrins that is relevant in cell adhesion [6,7,8,9,10]. However, the RGD motif has not been detected in the ENG sequence derived from rat [11], mouse [8], porcine [12] or chicken [13], nor in other protein counterparts. Notwithstanding, they do preserve the acidic residue (aspartic acid or glutamic acid) that is necessary for the interaction with the integrin β subunits [14,15].

The sequences of the transmembrane and cytoplasmic domains of ENG are highly conserved across species [8,9,10,11]. Moreover, these regions share a strong similarity with the transmembrane and cytoplasmic domains of betaglycan (71%) [16]. Within the cytoplasmic domain, a serine–methionine–alanine (SMA) tripeptide constitutes the C-terminal stretch of the protein, a sequence that can be considered a PSD-95/Discs-large/ZO1 (PDZ)-binding motif [17]. In fact, this motif has been demonstrated to mediate the interaction of ENG with the PDZ domain-containing protein, G alpha interacting protein (GAIP)-interacting protein C-terminus (GIPC), which regulates the retention of ENG at the endothelial cell (EC) surface [18,19].

The ENG messenger RNA (mRNA) is alternatively spliced, producing two isoforms that differ in the length of their cytoplasmic tail: 14 aa (S-ENG) or 41 aa (L-ENG) long [5]. Both isoforms have the first seven juxtamembrane residues of their cytoplasmic domains, although S-ENG lacks the PDZ-binding motif [5,7,18]. The intron located between exons 13 and 14, which contains a TAG stop codon, is spliced out of L-ENG mRNA, whereas it is retained by splicing factor-2 in S-ENG mRNA, allowing the generation of a shorter transcript [5,20]. The L-ENG is the isoform predominantly expressed in most tissues [5], although S-ENG might play key roles in some pathological contexts [21,22] as described later in this review.

## 3. The Physiological Role of Endoglin

Based on the aa sequence similarity between ENG and the TGFβ co-receptor betaglycan [16], the role of ENG in TGFβ signaling has been studied intensely, which has been crucial to elucidating its mode of action [1]. In fact, there is considerable evidence regarding ENG and its regulation of the TGFβ pathway.

The TGFβ signaling plays a crucial role in the proliferation, migration, differentiation, and apoptosis [23]. The human TGFβ superfamily comprises TGFβs (1, 2, and 3), bone morphogenic proteins (BMPs), activins (A and B), and growth differentiation factors (GDFs) [23]. Seven TGFβ type-I receptors (TGFβRI: activin-like kinases (ALKs) 1–7) and five TGFβ type-II receptors (TGFβRII) have been reported to be expressed in the human genome [23]. In the canonical signaling pathway, after binding of the ligand to TGFβRII, it activates and transphosphorylates TGFβRI, which subsequently transduces the signal by phosphorylating the downstream receptor-regulated SMAD (R-SMAD) molecules [23]. The involvement of different TGFβRI (ALKs) can provoke the induction of different R-SMADs [24]. Indeed, the activation of ALK5 triggers phosphorylation of SMAD2 and SMAD3, whereas activated ALK1 mediates SMAD1, SMAD5, and SMAD8 phosphorylation [24]. Phosphorylated SMAD 2/3 or 1/5/8 specifically associate with a common partner SMAD (co-SMAD) (SMAD4 in mammals) and these complexes are translocated to the nucleus, where they modulate the transcription of specific target genes [24,25,26,27,28]. In the case of non-canonical TGFβ signaling, several kinases can be activated, such as mitogen activated protein kinases (MAPKs), extracellular signal-regulated kinase (ERK), c-Jun N-terminal kinase (JNK), phosphatidylinositol-3-kinase (PI3K), or Rho-associated coiled-coil kinase (ROCK) [29,30].

ENG has been widely described to act as a co-receptor of the TGFβ cytokine superfamily [16]. It is known that ENG forms a heterodimeric receptor complex with TGFβRI and TGFβRII, modulating the ligand-receptor interaction [18]. Specifically, in association with TGFβRII ENG binds TGFβ1, TGFβ3, activin A and BMP-7, it binds to BMP-2 in the presence of TGFβRI, and it can also bind directly to BMP-9 or -10 [16,31,32,33]. Upon ligand binding, activated TGFβRII interacts with the extracellular and cytoplasmic domains of ENG [34]. At this point, the kinase activity of TGFβRII phosphorylates the cytoplasmic serine (Ser) 631 and 635 residues of ENG [18,34]. This association enhances the phosphorylation status of TGFβRI, which subsequently transduces the signal by phosphorylating R-SMADs [18,34]. Moreover, ENG interacts with TGFβRI through two interaction sites in the extracellular domain and one in the cytoplasmic domain [18,34]. TGFβRI phosphorylates ENG at threonine (Thr) residues and it is then released from the complex [35]. Interestingly, L-ENG can be phosphorylated at three different Ser/Thr phosphorylation sites 8-fold more strongly than S-ENG [35]. 

ENG plays an essential role during embryonic development, with ENG deficiency causing early embryonic lethality in mice [36,37]. Many studies have demonstrated that ENG is expressed in the mesoderm and that regulates hematopoiesis at early stages of gestation through the ALK1-SMAD1/5 pathway [38,39,40]. More specifically, ENG is required for the specification of hemangioblasts, embryonic progenitors of the hematopoietic and endothelial lineages, and for hematopoietic commitment, which is particularly important for proper myelopoiesis and erythropoiesis [39,40,41,42]. In line with these data, the hematopoietic and endothelial progenitors that emerge in close association during embryogenesis can be distinguished by their levels of ENG [43]. This regulation of ENG expression is associated with the modulation of TGFβ/BMP signaling, which ultimately controls definitive hematopoiesis [43]. Likewise, ENG regulates the divergence of cardiac and hematopoietic fate through BMP and Wingless/Integrated (WNT) signaling [44], and it is involved in heart septation and valve formation during early human development [45,46]. In this context, ENG is also necessary for correct adult erythropoietic development [47].

Given the essential roles of ENG during early development, it is no surprising that the expression of this protein is one of the criteria required to define multipotent mesenchymal stem cells (MSCs) [48]. In fact, ENG is crucial to determine the differentiation potential of several MSC populations (e.g., osteogenic, chondrogenic, adipogenic or myogenic) [49,50,51]. Indeed, ENG is downregulated over the time course of multi-lineage differentiation of human umbilical cord blood-derived MSCs [52,53].

In adults, ENG is predominantly expressed by active vascular ECs [1], as well as syncytiotrophoblasts [54,55,56], immature proerythroblasts [57], macrophages [58], bone marrow (BM) stromal cells [59], vascular smooth muscle cells (VSMCs) [60], MSCs [61], fibroblasts [62], mesangial cells [63], hematopoietic stem cells (HSCs) [64], chondrocytes [65], keratinocytes [66], periodontal ligament cells [50], hepatic stellate cells [11] and mast cells [67].

In terms of the modulation of ENG expression, its promoter is activated by TGFβ1 due to the binding of the Sp1 transcription factor [2,3]. Indeed, several studies demonstrated that TGFβ1 induces the expression of ENG in mesangial [63], HSC progenitor [64], VSMCs [60], hepatic stellate [68] and BM stromal [69] cells. Hypoxia is another well-known phenomenon that enhances ENG expression in the endothelium [70,71]. This mechanism protects ECs from apoptosis and it supports angiogenesis [70,71]. Similarly, angiotensin II upregulates ENG expression in human coronary artery ECs [72]. An opposing effect has been observed in the presence of the tumor necrosis factor α (TNFα), which downregulates the expression of ENG in the endothelium [73].

ENG has been described to be actively involved in regulating the balance between the TGFβ and BMP signaling in many of these cell types. 

In ECs, ENG promotes TGFβ/ALK1-induced SMAD1/5 responses and it indirectly inhibits the TGFβ/ALK5 signaling pathway [24], stimulating EC proliferation [74,75]. In this respect, ENG is expressed abundantly in actively proliferating ECs (wound healing, inflammation) [76,77,78,79,80], exerting a remarkable role in angiogenesis [36,45,79,81,82]. In line with these data, mutations in the ENG gene are responsible for hereditary hemorrhagic telangiectasia (HHT) type 1 [83,84], an autosomal dominant vascular disorder characterized by arteriovenous malformations and frequent hemorrhages [85]. 

Furthermore, the balance between TGFβ/ALK1/SMAD1/5/8 and TGFβ/ALK5/SMAD2/3 activation is also regulated by ENG in chondrocytes [86]. Similarly, ENG inhibits TGFβ1-induced ALK5/SMAD3 signaling [87] and favors the activation of the BMP-7/SMAD1/5 pathway in myoblasts [88].

There is considerable evidence that the cellular response to TGFβ1 is controlled by ENG. Overexpression of L-ENG, and to a lesser extent S-ENG, antagonizes TGFβ1-mediated inhibition of EC proliferation [82,83,84,85,86,87,88,89]. Likewise, ENG overexpression impairs the inhibitory effects of TGFβ1 on the proliferation of myoblasts [87,90], monocytic cells [18,91], keratinocytes [66] and fibroblasts [92]. Apart from modulating the effects of TGFβ1 on proliferation, ENG is necessary to mediate the inhibition of trophoblast migration and invasion stimulated by TGFβ1 [93]. Moreover, ENG downregulates TGFβ1-induced collagen synthesis in myoblasts [94].

Likewise, ENG plays an important role in modulating cell motility and invasion depending on the cell context. In fibroblasts, ENG impairs cell migration [95,96] in a non-SMAD dependent manner by regulating PI3K/protein kinase B (AKT) signaling [96]. Similarly, ENG inhibits the migration of keratinocytes [97], pericytes [98] and trophoblasts [93,99]. By contrast, ENG triggers the spreading of myoblasts and the migration of VSMCs [100,101]. Moreover, ENG promotes a pro-fibrogenic phenotype in hepatic stellate cells by enhancing the expression of vimentin, α-smooth muscle actin and connective tissue growth factor [68,102], which suggests ENG influences the migratory capability of these cells.

In ECs, ENG regulates focal adhesions and filamentous-actin (F-actin) cytoskeletal organization via binding to the LIM-domain containing zyxin and zyxin-related protein 1 (ZRP1), thereby inhibiting EC migration [103,104,105]. Likewise, the interaction of ENG with β-arrestin2 results in their co-internalization in endocytic vesicles, and the suppression of TGFβ1–induced ERK activation and EC migration [106]. Moreover, through its association with the α5β1 integrin, ENG mediates crosstalk between this integrin and the ENG/ALK1 complex, selectively augmenting SMAD1/5/8 phosphorylation [107]. This activation of the SMAD1/5/8 pathway suppresses EC migration and apoptosis, promoting capillary stability and angiogenesis [107]. In this line, the PDZ domain-containing GIPC interacts with and stabilizes ENG at the cell surface to potentiate SMAD1/5/8 signaling and inhibit EC migration [19]. This ENG/GIPC complex also mediates the recruitment and activation of PI3K and AKT at the cell membrane, thereby regulating capillary stability and cell survival during angiogenesis [108]. Although these data suggest an inhibitory influence of ENG on EC migration, Jin and colleagues have demonstrated that ENG controls EC migration during vessel remodeling by modulating vascular endothelial growth factor (VEGF)-A-induced VEGF receptor (VEGFR)-2 signaling in a cell autonomous manner [109]. Therefore, future efforts should clarify and integrate the multiple roles of ENG in the regulation of EC behavior and angiogenesis.

Among its other roles in ECs, ENG mediates nitric oxide (NO)-dependent vasodilatation by regulating endothelial nitric oxide synthase (eNOS) expression in a TGFβ/ALK5/SMAD2 dependent manner [110,111]. Moreover, via its RGD motif, endothelial ENG binds to integrins on vascular mural cells (α5β1) [112], leukocytes (α5β1) [14] and platelets (α5β1, αIIbβ3) [113], thereby favoring the adhesion of these cells to the endothelium. In this context, ENG stabilizes endothelial barrier function through its interaction with cell adhesion proteins (e.g., Ras homolog family member A (RhoA) protein) [114]. 

Recently, new roles for ENG have been described in physiological contexts. For instance, ENG is necessary to maintain correct hair follicle cycling and proper stimulation of hair follicle stem cell niches [115]. Furthermore, ENG mediates the regulation of the thermogenic gene program of beige adipocytes [116].

To date, most of the studies on ENG have focused on the predominant L-ENG isoform [5]. However, there is evidence that S-ENG plays an equally important role in endothelial and myeloid senescence [20,21,22]. 

Altogether, this information expands our knowledge of ENG and suggests that the last word about the physiological role of this protein has not yet been written.

## 4. Role of Endoglin on Tumor Cell Behavior

ENG is involved in a series of processes that directly affect tumor cell behavior [117]. Besides ENG expression in tumor vessels, some neoplasms also present high levels of ENG in tumor cells, including melanoma, renal cell carcinoma (RCC), leukemias, certain subtypes of sarcomas, and breast, ovarian, endometrial, and prostate cancer [117,118,119,120,121,122,123,124]. The role of ENG in tumor cells depends on the cell context, in some cases promoting tumor development and progression, and playing an important role in oncogenic signaling, whereas in other cases it has been associated with tumor suppression [117]. Below, we firstly summarize the recent evidence suggesting that ENG acts as a tumor promoter followed by data supporting that ENG might play a tumor suppressive role in some cancer types. 

ENG mainly regulates malignant phenotypes of cancer cells by modulating TGFβ/BMP signaling, yet it also acts through other SMAD-independent mechanisms [125]. Indeed, by promoting BMP signaling or by activating focal adhesion kinase (FAK) and PI3K pathways, ENG induces tumor plasticity in both Ewing sarcoma and melanoma [118]. Accordingly, ENG downregulation hinders invasiveness and abrogates tumor growth in preclinical models of Ewing sarcoma and melanoma [118]. Likewise, ENG promotes the epithelial–mesenchymal transition, and it stimulates spheroid formation and the migratory abilities of pancreatic cancer cells [126,127]. Indeed, RNA interference (iRNA)-based and competitive inhibitor-based blocking of ENG induces the differentiation of pancreatic cancer cells and reduces their tumorigenicity in vivo [128]. Moreover, this ENG inhibition sensitizes tumor cells to gemcitabine, a conventional chemotherapeutic drug [128].

In line with these data, several studies have reported an important role for ENG in tumor malignancy and resistance to therapy. In fact, an ENG-positive subpopulation in RCC xenografts possesses self-renewal ability, contributing to in vivo tumorigenicity and chemoresistance to gemcitabine [129]. Similarly, ENG expression is associated with drug resistance, poor differentiation, advanced disease stage, and a high rate of recurrence in ovarian cancer patients [130]. Consequently, ENG inhibition with small interfering RNA (siRNA) in ovarian cancer cells produces a less aggressive phenotype, with the induction of apoptosis and enhanced sensitivity to carboplatin in in vivo models [131]. Moreover, the TRC105 anti-ENG antibody inhibits metastatic spread, thereby improving overall survival (OS) in animal models of high-grade serous ovarian cancer [132]. Interestingly, high levels of ENG are also correlated with poor OS and progression free survival (PFS) in acute myeloid leukemia [133].

ENG is also upregulated in prostate cancer cells upon radiation and it promotes resistance to treatment [134]. Indeed, ENG mediates the DNA damage response and the metabolic adaptations to stress caused by irradiation by regulating its downstream target sirtuin 1 (SIRT1) [134]. Consequently, in synergy with radiation, TRC105 significantly reduces tumor growth in prostate cancer xenograft models [134]. 

Despite the evidence described above, ENG also plays an important role as tumor suppressor in certain cancer types. In fact, ENG suppresses prostate cancer cell migration and invasion via the ALK2/SMAD1 pathway, and it inhibits tumor growth and metastasis in vivo, suggesting that ENG is a negative regulator of prostate cancer [135,136,137,138,139]. In addition, ENG gene methylation was associated with the lack of ENG in human breast tumors and a poor clinical outcome [140]. Moreover, ENG overexpression in MDA-MB-231 breast cancer cells impaired migration and invasion, and reduced lung metastasis in vivo [140]. Furthermore, silencing of the ENG gene by epigenetic regulation appears to be a frequent event in both lung cancer and esophageal squamous cell carcinoma [119,141]. This lack of ENG expression was associated with a more invasive phenotype, supporting the role of ENG as a cell invasion- and tumor-suppressor gene in these cancer types [119,141].

All these data reveal that role of ENG in tumor cell behavior is dependent on cell context and must be exploited individually for each cancer.

## 5. Impact on the Tumor Microenvironment

### 5.1. Role of Endoglin in the Tumor Microenvironment

**Figure 2:**
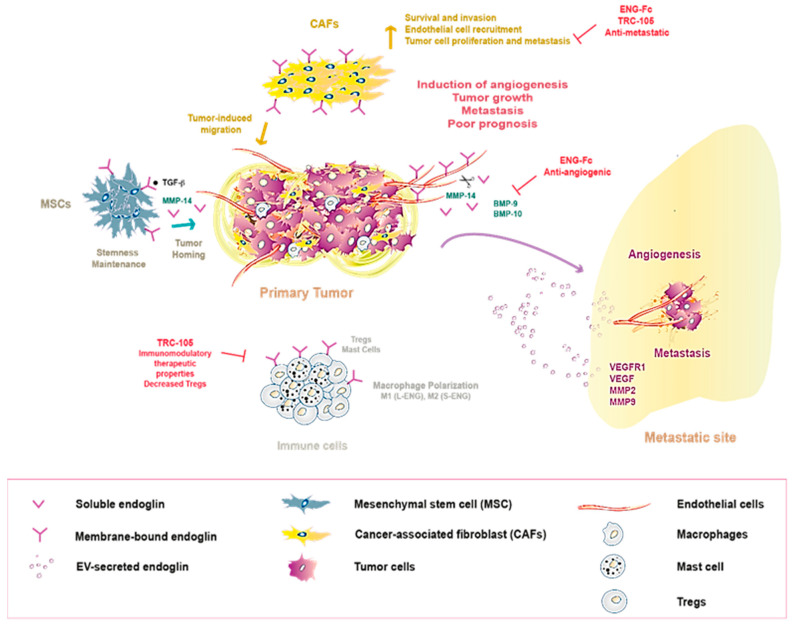
Endoglin acting in communication within the tumor microenvironment. Membrane-bound endoglin expression by several cell types within the tumor microenvironment (e.g., CAFs, macrophages, immune, and endothelial cells) and how its secretion (either soluble and/or in EVs) contributes to tumor progression and metastasis in different cancer models, making it a promising target for therapeutic strategies. Transforming growth factor β (TGFβ), bone morphogenic protein (BMP), matrix metalloproteinase 14 (MMP14), regulatory T cells (Tregs), extracellular vesicle (EV).

The tumor microenvironment (TME) plays a crucial role in cancer initiation and progression, a milieu that it is comprised of different cell types, including MSCs, fibroblasts, ECs, vascular cells, and immune cells [142]. The dynamic interplay between non-malignant cells and primary neoplastic cells is now well established, which contributes to tumor–stroma co-evolution and favors tumor promotion and metastasis [142,143,144]. ENG is present in some cell types that comprise the TME (e.g., ECs, MSCs, cancer-associated fibroblasts (CAFs), immune cells), actively regulating their behavior during tumorigenesis (Figure 2) [143]. 

As discussed above, ENG expression is specifically upregulated in actively proliferating ECs of tumor-associated vessels in different types of cancer, which has led to the use of ENG as a marker for tumor angiogenesis [124,145]. Accordingly, when ENG expression reflects a high intratumoral microvessel density (IMVD), this is closely correlated with a poor prognosis in pediatric adrenocortical tumors, pediatric rhabdomyosarcoma, astrocytomas and glioblastomas, hepatocellular (HCC) and oral squamous cell carcinomas, as well as in breast, lung, prostate, colorectal (CRC), ovarian, gastric, endometrial, esophageal, and head and neck and renal cancers [145,146,147]. Recently, a potential mechanism was proposed by which high endothelial ENG expression may be associated with a worse outcome of different solid tumor types [148]. It was shown that continuous ENG overexpression in mice promotes EC activation and triggers angiogenesis, although it impedes vessel stabilization and maturation, giving rise to more permeable vessels [148]. These alterations facilitate the intravasation of tumor cells and the subsequent development of metastases, leading to a poorer cancer prognosis [148].

There is significant evidence that ENG is an interesting target to block tumor angiogenesis. ENG downregulation inhibits the proliferation and angiogenesis of human ovarian carcinoma-derived ECs [149], and it decreases the number of tumor-associated vessels and the growth of mammary adenocarcinomas in vivo [150]. The growth of Lewis lung carcinoma xenografts is compromised in ENG haploinsufficient mice (Eng^+/−^), in which tumor vascularization is dampened relative to their wild-type (Eng^+/+^) littermates (as measured by capillary density, hemoglobin content, and vascular cell adhesion molecule-1 (VCAM-1) expression), in association with reduced eNOS phosphorylation [151]. Similarly, allelic ENG deletion slightly enhances the frequency of tumorigenesis, but it produces smaller, less vascularized, and less metastatic tumors in the transgenic adenocarcinoma mouse prostate (TRAMP) model [152]. Conversely, Anderberg and colleagues [153] showed that the ENG deficiency in mice (Eng^+/−^) facilitates tumor cell extravasation and increases metastatic spread. Although it is important to take into account that this model developed an HHT-like phenotype, which included vascular malformations and increased vascular permeability [153]. However, studies targeting ENG have mostly demonstrated good anti-angiogenic and anti-tumor effects to date [117,154,155,156] as explained below.

As mentioned previously, ENG is also a marker of MSCs [48,157] (Figure 2), and it plays a crucial role in the maintenance of their stem-like properties [158,159]. Moreover, ENG stimulates the homing of BM-derived MSCs to different tumor types through TGFβ signaling, including glioma, HCC, and breast cancer [160,161,162]. At tumor sites, MSCs can support tumor development and metastasis in several types of cancer [162,163,164,165]. Indeed, human ENG-positive MSCs are strongly correlated with disease progression and poor prognosis in gastric cancer [166], although this remains controversial since, in some studies, a suppressive effect of MSCs on the growth of leukemia and HCC has also been reported [167,168]. Therefore, the influence of ENG expression in MSCs and their precise role in cancer progression needs to be further explored.

ENG is also present in CAFs (Figure 2), specifically located at invasive borders of human CRC tumors, as well as in their lymph nodes and liver metastases, suggesting a role for ENG-expressing CAFs in CRC metastases [169]. In vitro experiments revealed that ENG is indispensable for BMP-9-induced signaling, and for CAF survival and invasion [169]. Moreover, ENG-expressing CAFs promote tumor cell invasion and metastasis in two pre-clinical (i.e., zebrafish and mouse) models of CRC metastasis [169]. Similarly, ENG is required for CAF viability and recruitment to prostate tumors [152]. Indeed, ENG expression in CAFs stimulates EC recruitment and proliferation of prostate cancer cells through a mechanism that involves secreted factors such as components of the insulin growth factor (IGF) signaling pathway [152]. In addition, a recent study showed that after androgen signaling deprivation therapy (ADT), ENG upregulation in a CAF population contributes to castration resistance-associated neuroendocrine differentiation of prostate tumor cells via a paracrine mechanism [170]. In keeping with these data, ENG targeting using the TRC105 anti-ENG antibody or an ENG ligand trap (ENG-Fc) significantly dampened metastatic spread in breast cancer mouse models, which was associated with a strong reduction in the CAF content of primary tumors [171]. Together, these findings further suggest ENG drives pro-tumoral and -metastatic behavior in CAFs, making it a potential target for CAF-directed therapy.

Some reports have shed light on the role of ENG on immune cells within the TME (Figure 2). ENG expression was detected in CD4+ T cells [172] and more specifically, ENG was seen to be expressed by a subset of CD4+ regulatory T cells (Tregs) in human and mouse CRC tumors [173]. Interestingly, the TRC105 significantly decreased the number of intratumoral Tregs in a CRC xenograft model, which contributed directly to its anti-tumor effects [173]. Similarly, treatment with TRC105 led to a significant decline in the number of Tregs in patients with metastatic urothelial carcinoma and castration-resistant prostate cancer [174,175], making Tregs a novel target for ENG targeted therapy. In addition, macrophages express ENG and require it to mediate their innate immune responses [58,176,177]. Remarkably, ENG can direct macrophages towards a tumor-inhibiting M1-like or tumor-promoting M2-like phenotype depending on the isoform expressed, L-ENG or S-ENG, respectively [21,178]. Further studies should be carried out to determine the predominant ENG isoform in the tumor-associated macrophages (TAMs) associated with different cancer types to clarify its precise contribution to an anti-tumor M1-like or pro-tumor M2-like phenotype. Recently, it was reported that ENG is expressed strongly in the cytoplasmic granules of human mast cells [67]. The intraclass correlation coefficient revealed that ENG is a reliable biomarker for these immune cells when compared with mast cell tryptase and toluidine blue [67]. Consequently, ENG may be involved in the pathogenic processes associated with mast cells and it may be useful in their diagnosis. Mast cells play an important role in modulating the TME and thus, in mediating tumor progression [179]. However, the function of ENG in tumor-associated mast cells has yet to be established.

In conjunction, these findings demonstrate that ENG expression in several cell types within the TME contributes to tumor progression and metastasis in different cancer models, making it a promising target for microenvironment-directed therapy (Figure 2). Recent data adds further support to this hypothesis [180], as targeting ENG-expressing cells in the TME (e.g., MSCs, TAMs, and ECs) significantly improves the efficacy of standard treatment with the anti-disialoganglioside GD2 antibody dinutuximab combined with activated natural killer cells against high-risk neuroblastomas [180].

### 5.2. Local and Distant Intercellular Communication by Secreted Endoglin (Exosomes and Soluble Endoglin)

Accumulating evidence reveals that tumors can communicate with their microenvironment through the secretion of specific molecules [181]. These secreted factors promote molecular and cellular changes in both neoplastic and TME cells, favoring tumor growth, pre-metastatic niche formation, and, ultimately, metastasis [181,182]. Soluble factors have been widely described as key players in this crosstalk [181].

A soluble form of ENG (Sol-ENG) has been identified that results from the shedding of the extracellular domain of membrane-associated ENG due to the activity of the matrix metalloproteinase (MMP)-14 [183]. Evidence suggests an anti-angiogenic function for Sol-ENG in cancer and, indeed, the ENG extracellular domain fused to an immunoglobulin Fc domain (ENG-Fc) that mimics Sol-ENG reduces in vitro and in vivo angiogenesis and inhibits tumor growth in a xenograft model of CRC [33,183] (Figure 2). This ENG-Fc binds specifically, and with high affinity to the TGFβ family ligands, BMP-9 and BMP-10, thereby blocking their downstream signaling [33]. In line with these data, ENG shedding appears to be an important mechanism by which TRC105 inhibits angiogenesis, as this anti-ENG antibody couples ENG to MMP14 in a complex at the cell surface, driving the release of the Sol-ENG anti-angiogenic factor [184].

So far, only a few in vitro studies have explored the involvement of Sol-ENG in cell-to-cell communication in cancer. Sol-ENG has been implicated in active crosstalk between BM-MSCs and breast cancer cells [185]. In this process, TGFβ1 secreted by malignant breast cancer cells acts through a paracrine mechanism on BM-MSCs to induce the production of soluble MMP14 [185]. In turn, the proteolytic activity of MMP14 releases the ENG at the breast cancer cell membrane, promoting their migratory potential by upregulating the SMAD2/3 pathway [185]. Along similar lines, BM-MSC-derived Sol-ENG protects myeloma cells from BMP-9-induced apoptosis [186]. The role of Sol-ENG in cancer is therefore controversial, since it seems to be involved in the inhibition of tumor-associated angiogenesis but also in activities that promote a malignant phenotype in myeloma and breast cancer cells. Moreover, elevated levels of Sol-ENG are correlated with poor prognosis in some cancer patients [187,188] as described below (Figure 2). Therefore, future studies should aim to elucidate the pro- or anti-tumoral effects of Sol-ENG on tumor-stroma crosstalk in different tumor types.

In addition to soluble factors, extracellular vesicles (EVs), such as exosomes (small EVS, 30–100 nm in size) and microvesicles (MVs, large EVs), are emerging as important mediators of TME communication [189,190]. Tumor-derived EVs contain biological information (RNA, DNA, proteins) from the primary tumor, and they can exert a functional influence once taken up by target cells, thereby modulating the TME and creating pre-metastatic niches, favorable microenvironments for metastasis to develop [190]. ENG-expressing MVs have been reported to participate in the induction of a pro-angiogenic and pro-metastatic microenvironment in human renal cancer [191] (Figure 2). These authors showed that ENG-expressing MVs derived from human renal cancer stem cells induce in vitro and in vivo angiogenesis, enhancing the organization, invasion, and resistance to apoptosis of ECs [191]. Moreover, these ENG-positive vesicles promote lung metastasis by upregulating the expression of different factors (VEGFR1, VEGF, MMP-2, MMP-9) that stimulate pre-metastatic niche formation in the lung [191] (Figure 2).

In this context, Yang and colleagues [192] demonstrated a mutual exchange of molecules between different MSC populations and tumor cells. More specifically, they showed that co-culture with ENG-expressing MSCs induces ovarian cancer and small cell hypercalcemic ovarian carcinoma-derived cells to acquire ENG, cells that barely express basal ENG [192]. These changes could occur through different mechanisms, such as exosome secretion and their subsequent cellular uptake [192]. However, the role of EV-secreted ENG is still not well understood and further studies in other tumor types should be performed to analyze the different molecular mechanisms through which ENG-expressing EVs could modulate the TME.

## 6. Clinical Implications of Endoglin

### 6.1. State-of-the-Art Endoglin-Based Therapies

As previously mentioned, ENG was initially described as a potent marker of angiogenesis [145], although in recent years it has also emerged as a promising drug target [117,156,169,171,173,174,175]. Therapies targeting ENG are based on evidence that this protein is mainly present in tissues undergoing some degree of repair or damage [193,194,195]. As described above, ENG is expressed strongly in proliferating ECs but also in cancer cells from different types of tumors [118,120,121,123]. Therefore, the generation of drugs to block this TGFβ co-receptor could represent an alternative anti-angiogenic and anti-tumor therapeutic strategy for neoplasms that are resistant to other anti-angiogenic agents (e.g., anti-VEGF) or chemotherapy.

The ENG-based therapies include the use of: (1) monoclonal antibodies; (2) antibody-drug conjugates (ADCs); (3) radiolabeled ENG antibodies; (4) ENG-based vaccines [117,175,196,197,198,199,200,201,202,203,204,205]. In general, the use of neutralizing monoclonal antibodies is an interesting therapeutic approach to target specific transmembrane receptors [206]. TRACON pharma developed the TRC105 monoclonal antibody, designed to block human ENG and to disrupt its activity as a TGFβ co-receptor, competing with BMP-9 [117]. Carotuximab (TRC105), induces antibody-dependent cellular toxicity of angiogenic ECs and ENG-expressing tumor cells [117]. This drug is currently undergoing phase I/II/III clinical trials to treat RCC, prostate cancer, HCC, advanced angiosarcoma, non-small-cell lung cancer, ovarian cancer, and glioblastoma as well as other advanced cancers [175,196,197,203,207]. Notably, anti-VEGF therapies, such as bevacizumab, specifically target VEGF and block angiogenesis [208]. However, cells can become resistant to this therapy in hypoxic conditions by switching to VEGF-independent angiogenesis through ENG overexpression [171,209]. Therefore, TRC105 could help to sensitize these tumors resistant to VEGF inhibition.

Clinically, the first in-human study with TRC105 was carried out on 50 patients with advanced refractory disease including sarcoma and CRC, prostate, renal, lung, breast and ovarian cancer [200]. No adverse events (AEs) were generally associated with this therapy and the drug was tolerated at a dose of 10 mg/kg/week or 15 mg/kg every two weeks [200]. This therapy was especially effective in one case of castrate-resistant prostate cancer and another patient with metastatic uterine-carcinosarcoma [200]. A reduction in cancer antigen (CA)-125 levels was also observed in ovarian cancer patients [200]. Moreover, the evaluation of circulating biomarkers associated with angiogenesis in serum samples from patients showed that TRC105 modulated the levels of VEGF and Sol-ENG [200].

The use of TRC105 was also studied in a phase II trial involving HCC patients who had progressed following prior treatment with the anti-angiogenic tyrosine kinase inhibitor, sorafenib [196]. Toxicity was acceptable with epistaxis and headaches as the most common AEs [196]. Unfortunately, TRC105 did not exhibit an appreciable clinical response in these patients when used as a single agent [196]. Thus, efforts have focused on the use of TRC105 in different combinations to increase efficacy and prevent resistance. A phase Ib study tested the combination of TRC105 with the anti-VEGF antibody bevacizumab in a total of 38 patients including: one esthesioneuroblastoma, 2HCC, 17 CRC, 11 ovarian, two renal, two lung, one cervical, one endometrial, and one peritoneal cancer [198]. The combination therapy was well tolerated and exhibited promising efficacy with partial responses (PRs) and stable disease (SD) in 45% of patients, 10 of whom had progressed after previous treatment with anti-VEGF monotherapy [198]. Interestingly, the evaluation of soluble biomarkers showed an increase in Sol-ENG and modulation of TGFβ1 in patients treated with TRC105 [200]. Patients with a clinical response (SD/PR) had low basal levels of ECM proteins like epithelial cadherin (E-cadherin), intercellular adhesion molecule 1 (ICAM-1) and osteopontin (OPN) after 3 weeks of treatment, as detected by enzyme-linked immunosorbent assay (ELISA) in plasma from CRC, ovarian, HCC, renal, lung, cervical, endometrial, neuroblastoma, and peritoneal cancer patients [200].

The combination of TRC105 with some small molecule inhibitors of VEGF has also been explored. For example, TRC105 in combination with sorafenib was evaluated in a phase I/II clinical trial including 25 patients with HCC [207]. This study was supported by pre-clinical data showing that resistance to sorafenib was associated with upregulation of ENG and that the addition of TRC105 to sorafenib led to enhanced tumor growth inhibition in preclinical models [207]. Results from this trial revealed that the combination was well-tolerated and was associated with promising outcomes, with an overall response rate (ORR) of 21% [207]. Increases in serum Sol-ENG were observed following TRC105 treatment as demonstrated elsewhere [207]. An initial phase Ib/IIa trial investigated the combination of TRC105 with pazopanib in patients with advanced soft tissue sarcoma, five of whom had angiosarcoma [210]. Angiosarcoma is a rare and aggressive endothelial tumor with poor prognosis [211]. Therapies targeting VEGF and other endothelial-related proteins have shown some promising results in combatting this tumor type [211]. There was preliminary evidence of clinical activity of combined TRC105/pazopanib treatment in 2/5 patients [210]. Given these encouraging results, a multicenter phase III trial exploring the efficacy of TRC105/pazopanib in patients with advanced angiosarcoma was set-up and is ongoing at the time of writing (TAPPAS: NCT02979899) [6]. Moreover, the combination of TRC105 and the VEGFR inhibitor axitinib was evaluated in a phase Ib trial involving patients with metastatic renal cell carcinoma (mRCC) who were refractory to first-line treatments [212]. This combined therapy showed a favorable toxicity profile and led to promising responses with a PR of 29% [212]. This preliminary evidence of activity led to a multicenter phase II trial enrolling 150 mRCC patients, a trial that was ongoing at the time of this writing (NCT01806064) [7]. In addition, TRC105 is currently being tested in combination with chemotherapy (paclitaxel/carboplatin) or bevacizumab in a phase Ib trial involving non-squamous cell lung cancer patients (NCT02429843) [6]. Another trial evaluating the safety and efficacy of the combination of TRC105 with other chemotherapeutic agents (letrozole/everolimus) is currently ongoing on patients with advanced hormone receptor positive and human epidermal growth factor receptor 2 (HER2) negative breast cancer (NCT02520063) [5].

The generation of ADCs is a promising field with a wide variety of options. ADCs are composed of three parts: (1) an antibody, specifically designed to bind to a cell surface target, and to induce internalization and intracellular uptake; (2) a payload, a drug that will be liberated into the cytoplasm after receptor-mediated endocytosis; together bound by (3) a chemical linker that brings stability to the structure and that allows the drug to be released once it is internalized [206,213,214]. The major advantage of ADCs is their high specificity, as only cells expressing the targeted protein are those that are likely to incorporate the drug [206,213,214].

Several studies indicate that anti-ENG ADCs may represent a promising clinical approach to effectively reduce tumor angiogenesis and/or induce cell death upon their binding to target-expressing tumor cells [214]. Our group has shown that treatment with anti-ENG ADCs strongly impaired tumor growth in Ewing sarcoma cell line-derived xenografts and patient-derived xenografts [215]. We found that anti-human ENG monoclonal antibodies alone had no effect on tumor growth [215]. Nonetheless, the conjugation of these antibodies to a cytolysin or nigrin B payload led to a dramatic reduction in tumor size and recurrence when compared to the chemotherapeutic agent irinotecan alone [215]. Notably, the expression of ENG was heterogeneous in human Ewing sarcoma neoplastic clinical samples, which stresses the need for a good screening procedure (ENG expression) in a clinical context [215].

Antibodies targeting ENG also represent an attractive strategy for imaging, specifically through their binding to active endothelium [201,202,216]. An interesting experiment explored the biodistribution of TRC105 conjugated with gold nanoparticles (AuNPs) in mice bearing ENG-expressing melanoma xenografts [201]. These immunoconjugates were taken up strongly and specifically by melanoma tumors, with preserved tumor contrast in positron emission tomography (PET) imaging [201]. Moreover, no major tracer accumulation was detected over time in non-specific organs [201]. Hence, this study supports the use of anti-ENG antibodies conjugated with AuNPs for cancer imaging and lays the basis for further theragnostic applications.

Finally, DNA vaccines encoding ENG have been reported to be useful in terms of immune system activation [204,217]. In 2006, it was shown how an oral DNA vaccine encoding ENG could suppress metastatic spread in the D2F2 breast carcinoma mouse model [217]. Treatment with the vaccine induced activation of antigen-presenting dendritic cells and CD8+ T cells against ENG-positive target cells [217]. The immune responses caused by the vaccine were proposed to halt metastatic dissemination by eliminating proliferating ECs in the tumor vasculature [217]. More recently, it was demonstrated that the combination of an ENG-based DNA vaccine with immunomodulatory agents, such as cyclophosphamide or interleukin (IL)-12, exerted potent prophylactic and therapeutic anti-tumor activity in mouse models of aggressive melanoma [204]. Interestingly, this therapy led to polarization of the TME with a reduction of Tregs and inhibition of angiogenesis [204].

### 6.2. Soluble Endoglin, a Promising Biomarker in Liquid Biopsies

As described previously, ENG can be shed from the cell membrane as Sol-ENG [183]. This soluble form of ENG has been detected in the plasma and other body fluids from patients with several types of solid tumors [187,188,218,219,220] or with myeloid hematopoietic malignancies [221]. High levels of Sol-ENG are correlated with poor survival, relapse, and metastatic disease in different types of cancer such as CRC, prostate, breast, and lung cancer [187,188]. Interestingly, the levels of Sol-ENG increase after treatment with TRC105 in cancer patients, which may reflect the direct anti-angiogenic effects of the drug [200]. These findings suggest that Sol-ENG could also be used as a biomarker of response to therapy. In this respect, it is important to remember that the role of Sol-ENG in cancer remains unclear, and that although its levels are correlated with poor outcomes, it has also been described to play an important role in the inhibition of tumor angiogenesis [33,183]. Therefore, more research is needed to clarify the effects of Sol-ENG in tumor cells and in the TME.

There is evidence that Sol-ENG also plays a relevant role in pre-eclampsia [222,223,224,225], a condition characterized by maternal hypertension and proteinuria, representing a major cause of maternal/fetal mortality [226]. In pre-eclampsia, excess circulating levels of anti-angiogenic and pro-coagulation factors lead to placental endothelial dysfunction, vascular permeability and ischemia [226]. More specifically, Sol-ENG contributes to the manifestation of pre-eclampsia by blocking pro-angiogenic factors, inhibiting vessel formation, and increasing permeability [225]. Indeed, pregnant women with pre-eclampsia have significantly higher levels of Sol-ENG, which later revert to basal levels after birth [227]. Likewise, EVs expressing ENG have been proposed as circulating biomarkers for endothelial injury and pre-eclampsia [228,229].

## 7. Conclusions

In conclusion, our understanding of the function of ENG in tumor cells and in the TME has progressed in recent years. Besides being an important mediator of tumor cell behavior in several cancer types, there is increasing evidence of a crucial role for ENG in modulating the TME, making it favorable for tumor progression and metastatic spread. Thus, apart from classic anti-angiogenic drugs, anti-ENG therapies are emerging as a promising approach for multi-target directed cancer therapy. There are a growing number of clinical trials testing the combination of the TRC105 anti-human ENG antibody with standard therapies in different tumor types, including chemotherapeutic agents (paclitaxel or carboplatin) or VEGF inhibitors (bevacizumab, sorafenib, or pazopanib). Interestingly, preliminary evidence shows clinical activity of such combinations in several patients who were refractory to first-line treatments, suggesting that combination treatments could overcome resistance to therapy. Moreover, recent studies show that other ENG-based therapies like anti-ENG ADCs or DNA vaccines encoding ENG could represent potential therapeutic options for cancer patients, enhancing tumor cell specificity and immune system activation, respectively. Future research should aim to explore the precise contribution of ENG expression in specific cell types to tumor progression and metastatic spread using cell-type specific ENG knockout or overexpressing mouse models. To sum up, novel combinations of anti-ENG therapies with current first-line treatments should be tested in pre-clinical models with the goal of bringing these advances more rapidly to the clinic. This will require worldwide, well-designed clinical trials involving patients who are refractory to standard anti-angiogenic treatments.

## Data Availability

Not applicable.

## Abbreviations

aa	Amino acids
ADCs	Antibody-drug conjugates
ADT	Androgen signaling deprivation therapy
AEs	Adverse events
AKT	Protein kinase B
ALK	Activin-like kinase
AuNPs	Gold nanoparticles
BM	Bone marrow
BMP	Bone morphogenic protein
CA	Cancer antigen
CAF	Cancer-associated fibroblast
co-SMAD	Common partner SMAD
CRC	Colorectal cancer
Cys	Cysteine
EC	Endothelial cell
E-cadherin	Epithelial cadherin
ECM	Extracellular matrix
ELISA	Enzyme-linked immunosorbent assay
ENG	Endoglin
eNOS	Endothelial nitric oxide synthase
ERK	Extracellular signal-regulated kinase
EV	Extracellular vesicle
F-actin	Filamentous-actin
FAK	Focal adhesion kinase
GAIP	G alpha interacting protein
GDF	Growth differentiation factor
GIPC	GAIP-interacting protein C-terminus
HCC	Hepatocellular carcinoma
HER2	Human epidermal growth factor receptor 2
HHT	Hereditary hemorrhagic telangiectasia
HSC	Hematopoietic stem cell
ICAM-1	Intercellular adhesion molecule 1
IGF	Insulin growth factor
IL	Interleukin
IMVD	Intratumoral microvessel density
iRNA	RNA interference
JNK	c-Jun N-terminal kinase
MAPKs	Mitogen activated protein kinases
MMP	Matrix metalloproteinase
mRCC	Metastatic renal cell carcinoma
mRNA	Messenger RNA
MSC	Mesenchymal stem cell
MVs	Microvesicles
NO	Nitric oxide
OPN	Osteopontin
ORR	Overall response rate
OS	Overall survival
PDZ	PSD-95/Discs-large/ZO1
PET	Positron emission tomography
PFS	Progression free survival
PI3K	Phosphatidylinositol-3-kinase
PR	Partial response
RCC	Renal cell carcinoma
RGD	Arginine-glycine-aspartic acid
RhoA	Ras homolog family member A
ROCK	Rho-associated coiled-coil kinase
R-SMAD	Receptor-regulated SMAD
SD	Stable disease
Ser	Serine
siRNA	Small interfering RNA
SIRT1	Sirtuin 1
SMA	Serine-methionine-alanine
Sol-ENG	Soluble endoglin
Sp1	Specificity protein-1
TAM	Tumor-associated macrophage
TGFβ	Transforming growth factor β
TGFβRI	TGFβ type-I receptor
TGFβRII	TGFβ type-II receptor
Thr	Threonine
TME	Tumor microenvironment
TNFα	Tumor necrosis factor α
TRAMP	Transgenic adenocarcinoma mouse prostate
Treg	Regulatory T cell
VCAM-1	Vascular cell adhesion molecule-1
VEGF	Vascular endothelial growth factor
VEGFR	Vascular endothelial growth factor receptor
VSMC	Vascular smooth muscle cell
WNT	Wingless/Integrated
ZP	Zona pellucida
ZRP1	Zyxin-related protein 1
